# Variation in intraocular pressure by sex, age, and geographic location in China: A nationwide study of 284,937 adults

**DOI:** 10.3389/fendo.2022.949827

**Published:** 2022-08-25

**Authors:** Xuan Liu, Xue Pan, Yuan Ma, Cheng Jin, Bo Wang, Yi Ning

**Affiliations:** ^1^ Beijing Tsinghua Changgung Hospital, School of Clinical Medicine, Tsinghua University, Beijing, China; ^2^ Department of Ophthalmology, Beijing Shijitan Hospital, Capital Medical University, Beijing, China; ^3^ Peking University Health Science Center, Meinian Public Health Research Institute, Beijing, China; ^4^ School of Public Health, Hainan Medical University, Haikou, China

**Keywords:** intraocular pressure (IOP), sex, age, geographic distribution, reference interval

## Abstract

**Objective:**

To investigate the distribution characteristics of intraocular pressure (IOP) by sex, age, and geographic location in China and to build the corresponding reference intervals (RIs).

**Material and methods:**

A cross-sectional, multi-centered, population-based study was conducted. All data were collected from participants without eye diseases who underwent ophthalmological examinations in 170 Health Screening Centers in mainland China in 2018. The non-contact tonometer was used to measure IOP. The age-, sex-, and province-specific RIs of IOP were investigated. The IOP of different age–sex groups was further explored by stratifying according to height, body mass index (BMI), blood pressure, altitude, and geographic area.

**Results:**

During the study, a population-based sample of 284,937 participants was included. The distribution of measured IOP followed an approximately Gaussian distribution, with a higher mean value in men than in women. The IOP showed a general trend of decline with age for both men and women and varied across geographical locations. The mean IOP was 15.4 (95% CI: 9.1-21.6) mmHg for men and 14.9 (95% CI: 9.0-20.8) mmHg for women. For men, it decreased from 11.0-23.5 mmHg at age 18-24 years to 10.5-20.5 mmHg at age ≥70 years. For women, it decreased from 10.5-22.0 to mmHg at age 18-24 years to 10.0-21.0 mm Hg at age ≥70 years.

**Conclusions:**

The IOP varied with age, sex, metabolic disorders and geographic location. These RIs should be considered in the clinical process of glaucoma diagnosis and treatment.

## Introduction

Glaucoma is the most common cause of irreversible blindness worldwide (1, 2), with the number of patients expected to reach 111.8 million by 2040 (2). From a pathophysiological and therapeutic point of view, intraocular pressure (IOP) is the primary modifiable risk factor, since progression of glaucoma usually stops if IOP is lowered by 30-50% from baseline (3). Assessment of IOP helps to make decisions for prevention, diagnosis, and treatment of glaucoma.

The prevalence of glaucoma varies with sex (4, 5), age (4, 6, 7), and across geographic regions (2, 8, 9). Glaucoma prevalence varies from 2.93% in Europe to 3.40% in Asia and 4.79% in Africa (2). Previous studies have defined a normal range of IOP as 10 to 21 mmHg (10). However, participants in these studies were Caucasians, and the normal reference interval (RI) may be different for Asians. An ophthalmologically normal Japanese population-based study reported an average IOP of 14.1 ± 2.3 mmHg (11). Additionally, a positive correlation between age and IOP in Caucasians (12), and an inverse correlation between age and IOP in Japanese have been reported (11). Although a few studies have examined the variation of IOP by age, gender, and geographic location across China (13, 14), they did not capture the heterogeneity due to relatively small sample sizes. One study (15) investigated the association of IOP with age and sex; however, geographic distribution and metabolic factors were ignored. In order to investigate the distribution characteristics of IOP and to provide specific RI, we conducted this study using available data from 170 Screening Centers located across the majority of provinces in mainland China.

## Methods

### Study population

The Meinian OneHealth Group is a public company with health screening centers located in most provinces in mainland China, which provide annual or periodic health examinations for their members. All screening equipment for eye examinations were approved by the authorities. All participants (aged ≥18 years) underwent a brief interview about demographic information, medical history, and health checkup.

**Figure 1:**
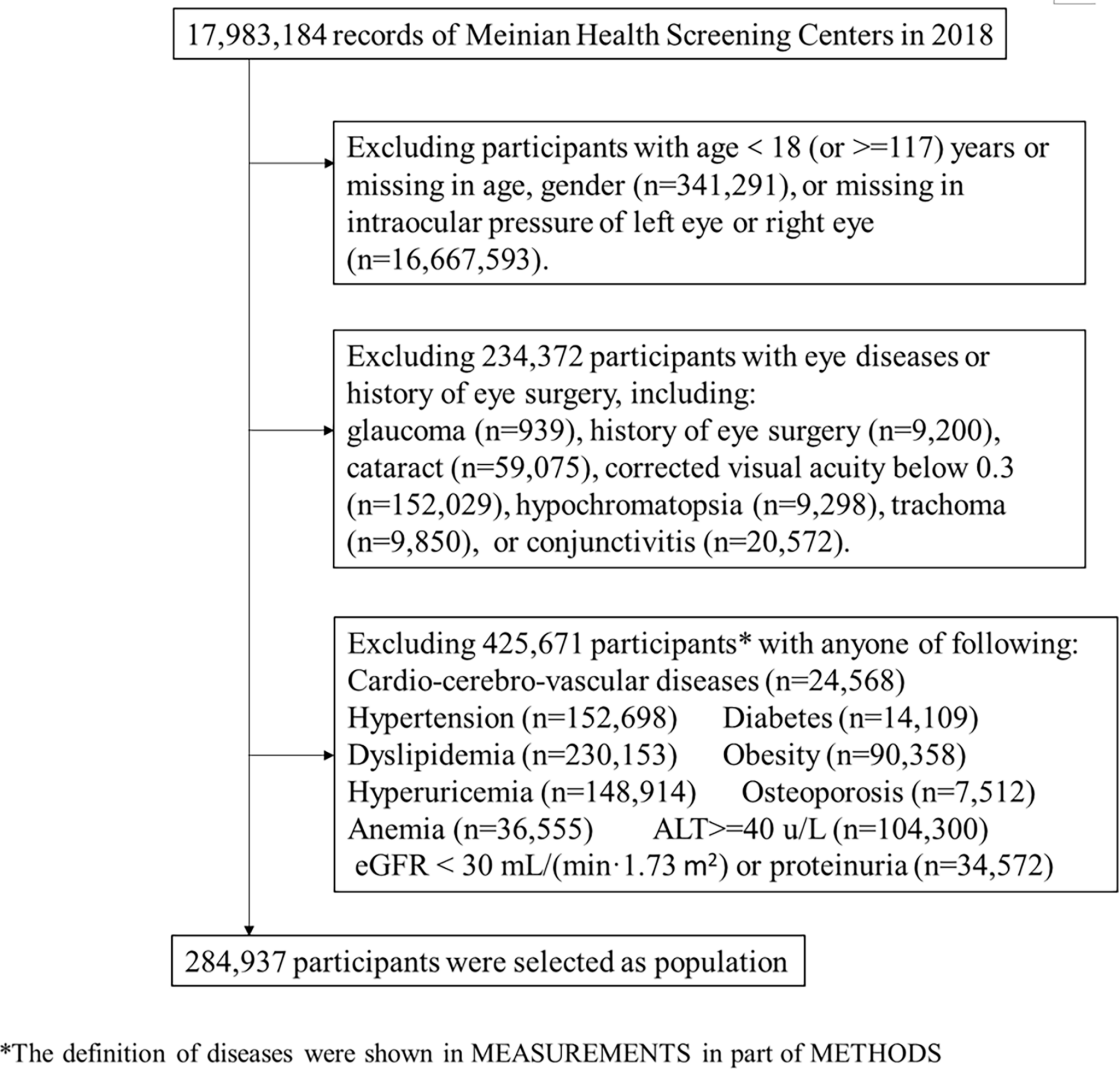
Flowchart of selection of the study participants.

Participants (n=17,983,184) underwent ophthalmological examinations (including visual acuity, IOP measurements, slit-lamp examination, and fundus photography) in 267 screening centers from January 1^st^ to December 31^st^, 2018. We included 974,300 participants aged ≥18 years with no missing values for age, sex, and intraocular pressure of both eyes. We excluded 234,372 participants with previously diagnosed glaucoma, history of eye surgery (including cataract surgery, retinal detachment surgery, vitrectomy, corneal refractive surgery), cataract, trachoma, hypochromatopsia, conjunctivitis, or corrected visual acuity below 0.3. To explore the IOP RI for healthy people, we further excluded 425,671 participants with certain diseases, including cardio-cerebro-vascular diseases, hypertension, diabetes, dyslipidemia, obesity, hyperuricemia, severe hepatic disease, severe renal disease, osteoporosis, and anemia (detailed diagnostic criteria described in the measurement of covariates). For those who attended two screenings or more, results from the most recent checkup were included to ensure the independency of data. Therefore, we included 284,937 participants from 170 health screening centers across 81 cities in mainland China in our study (**Figure 1**).

This study complies with the tenets of the Declaration of Helsinki, and institutional review board (IRB)/Ethics Committee approval was obtained. This study was approved by the Ethics Committee of Beijing Tsinghua Changgung Hospital, affiliated with Tsinghua University (Ethics number 18181-0-01). All participants provided informed consent. Data were centrally managed and stored. Individual identifiers were removed and remained anonymous during the entire study process.

### Measurements

#### Measurements of IOP

Ophthalmic examination consisted of slit-lamp examination (cornea, lens, iris, and aqueous), non-mydriatic fundus photography, and IOP measurement. The IOP was measured with an auto non-contact tonometer (HNT-7000 Non-Contact Tonometer made in Korea, Reichert 7CR Auto Non-Contact Tonometer made in USA, or Topcon CT-80A Non-Contact Computerized Tonometer made in Japan), and all procedures were performed strictly according to instructions and unified operation specifications.

#### Measurements of covariates

All laboratories in this study successfully completed a standardization and certification program. Blood samples were drawn by venipuncture after 8–12 h of overnight fasting. Fasting blood glucose (FBG), total cholesterol (TC), triglyceride (TG), high-density lipoprotein (HDL), low-density lipoprotein (LDL), creatinine (Cr), alanine transaminase (ALT), and aspartate transaminase (AST) were measured using automatic biochemical analyzers with commercially available reagents at the clinical biochemical laboratories in each center.

Estimated glomerular filtration rate (E-GFR) was calculated according to the Chronic Kidney Disease Epidemiology Collaboration creatinine equation (16), and severe chronic kidney disease was defined as eGFR less than 30 ml/min/1.73 m ^2^ or with proteinuria. Diabetes was defined as a self-reported physician-diagnosis history, currently treated with insulin or oral hypoglycemic agents, or *via* FBG concentrations ≥7.0 mmol/L. Dyslipidemia was defined as a self-reported dyslipidemia history, TC ≥6.22 mmol/L, TG ≥2.26 mmol/L, HDL <1.04 mmol/L, or LDL ≥4.92 mmol/L. Hyperuricemia was defined as a uric acid concentration of ≥360 µmol/L in women and ≥420 µmol/L in men. Severe hepatic insufficiency was defined as an ALT concentration ≥40 U/L. Hemoglobin levels were measured *via* blood cell analysis (5-Part Differential), and levels <120 g/L in men or <110 g/L in women was regarded as anemia.

Left ventricular hypertrophy, atrial flutter, or atrial fibrillation was diagnosed by electrocardiogram. Severe valvular heart disease was diagnosed by echocardiography, including mitral valve stenosis (mitral valve area <1.5 cm^2^), mitral regurgitation (regurgitant fraction ≥30%), aortic stenosis (aortic valve area <1.0 cm^2^), aortic regurgitation (regurgitant fraction ≥30%), tricuspid valve stenosis, tricuspid regurgitation, pulmonary valve stenosis, and pulmonary valve regurgitation. Stroke was diagnosed by cranial computed tomography or magnetic resonance imaging. Carotid artery stenosis was diagnosed by carotid color Doppler ultrasound.

Obesity was defined as a body mass index (BMI) of 28 kg/m^2^ or higher. Systolic blood pressure (SBP), diastolic blood pressure (DBP), and heart rate (HR) were measured using a digital automatic blood pressure (BP) monitor after participants were seated for at least 5 min. The mean values of the two BP and HR readings were recorded. Hypertension was defined as a self-reported physician-diagnosis history, currently treated with antihypertensive agents. Prehypertension was defined as 120 mm Hg ≤ SBP <140 mmHg or 80 mm Hg ≤ DBP <90 mmHg without a history of hypertension. Tachycardia was defined as an HR >100 beats/min and bradycardia was defined as an HR of <60 beats/min. Data on gross domestic product per capita (GDP) were collected from the China Statistical Yearbook for 2018 (17).

### Statistical analysis

The IOP reported for the participants was the mean value of IOP in the left and right eyes without specific indication. As the distribution of IOP differed between men and women, we performed separate analyses between sexes. Besides age, sex, and geographical regions, the distribution of IOP according to height, BMI, blood pressure, altitude, and north or south geographic area were also explored. Height and altitude were divided by tertiles, and geographic area was divided by latitude of 33°N.

To describe IOP distribution, the mean and 95% confidence interval (mean ± 1.96 SD) were calculated, where SD denotes standard deviation. The medians (2.5^th^-97.5^th^ percentiles) were also presented, which were used as the reference intervals (RIs) in this study, since the Clinical and Laboratory Standards Institute (18) recommends the non-parametric computation of a 0.95 coverage interval. The analysis of variance and the SNK test were applied to compare IOP levels among different age groups (the rank of IOP values were used when median values were compared). A general linear model was constructed to estimate age-GDP-adjusted IOP mean values. Choropleth maps were produced by R software to visually examine the geographical variation in IOP. Statistical analyses were conducted using SAS software (SAS, Inc., Cary, NC, USA, version 9.4) and R software (version 3.6.1). A p-value of <0.05 was considered statistically significant.

## Results

### Basic characteristics and IOP distribution for all participants

**Figure 2:**
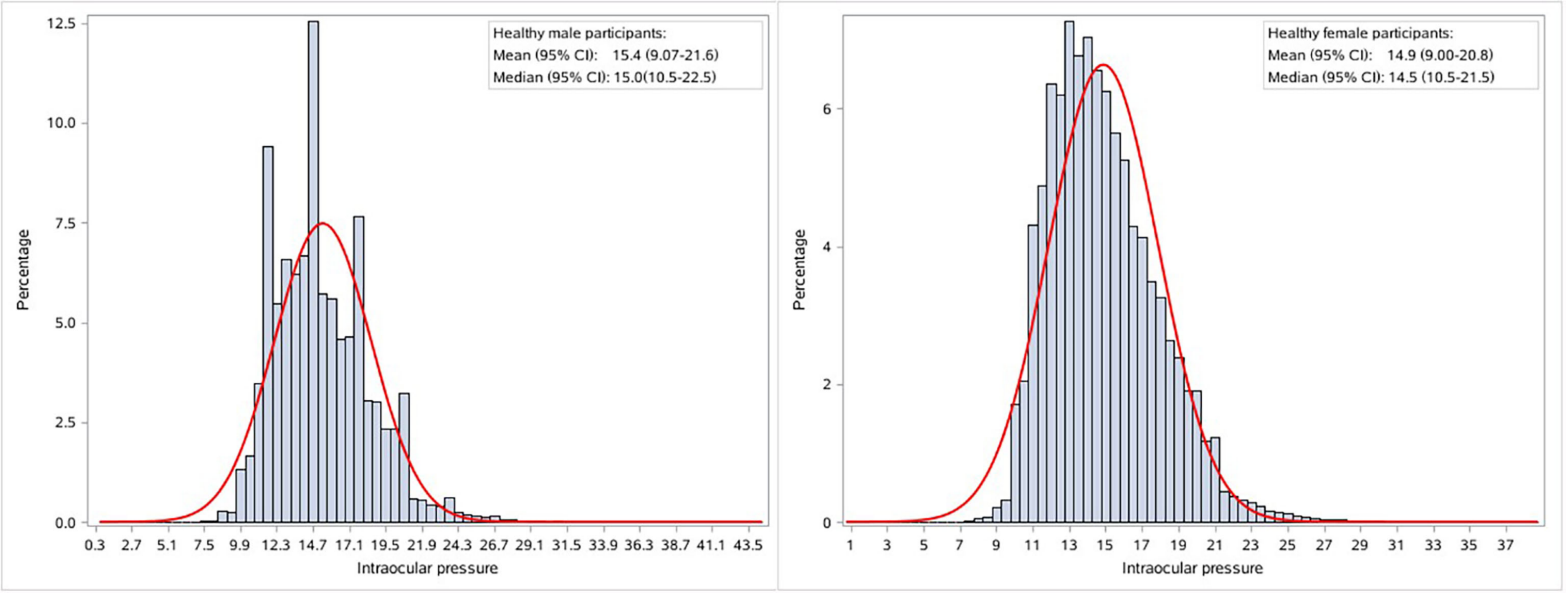
Sex specific distribution of intraocular pressure in healthy participants. The red curve denotes the normal distribution density curve.

Among the 284,937 participants included, approximately 33.9% were men. The average age was 39.9 ± 11.9 years (mean± SD) for men and 38.7 ± 10.7 years for women (**Table 1**). The mean IOP was 15.4 (95% CI: 9.1-21.6) mmHg for men and 14.9 (95% CI: 9.0-20.8) mmHg for women. A higher proportion of men than women were prehypertensive, prediabetic, and overweight. The distribution of IOP followed an approximately Gaussian distribution, with a slight right-ward skew in both sexes (**Figure 2**).

**Table 1:** Sociodemographic and clinical characteristics of study participants (N=284,937) by sex.

	Men	Women
N	96,517	188,420
Age (years), Mean ± SD	39.93 ± 11.91	38.66 ± 10.66
<30, n (%)	21,292 (22.1)	41,772 (22.2)
30-39, n (%)	32,504 (33.7)	69,050 (36.6)
40-49, n (%)	20,028 (20.8)	44,129 (23.4)
50-59, n (%)	15,352 (15.9)	25,402 (13.5)
60-69, n (%)	6,611 (6.8)	7,571 (4.0)
>=70, n (%)	730 (0.8)	496 (0.3)
IOP (mmHg), Mean ± SD	15.35 ± 3.20	14.88 ± 3.01
Right IOP	15.26 ± 3.37	14.80 ± 3.16
Left IOP	15.44 ± 3.40	14.95 ± 3.20
Gross Domestic Product per capita (CNY), n (%)		
Quintiles 1	19,935 (20.7)	39,855 (21.2)
Quintiles 2	20,160 (20.9)	34,252 (18.2)
Quintiles 3	27,515 (28.5)	63,625 (33.8)
Quintiles 4	9,551 (9.90)	12,913 (6.85)
Quintiles 5	19,356 (20.1)	37,775 (20.0)
Hypertension, n (%)		
Normal	49,961 (53.0)	133,373 (72.7)
Prehypertension	44,335 (47.0)	50,163 (27.3)
Diabetes, n (%)		
Normal	15,004 (77.2)	30,324 (83.3)
Prediabetes	4,422 (22.8)	6,099 (16.7)
BMI^†^ (kg/m^2^), Mean ± SD	22.79 ± 2.55	21.79 ± 2.51
<18.5, n (%)	4,989 (5.35)	16,150 (8.92)
<24, n (%)	56,586 (60.7)	128,591 (71.1)
<28, n (%)	31,699 (34.0)	36,230 (20.0)
Height (cm), n (%)		
Tertile 1	4,737 (5.08)	109,019 (60.2)
Tertile 2	31,123 (33.4)	63,965 (35.4)
Tertile 3	57,416 (61.6)	7,989 (4.41)
City size*, n (%)		
Small or medium sized city	12,513 (13.0)	28,075 (14.9)
Large city	42,899 (44.4)	80,922 (42.9)
Mega city and above	41,105 (42.6)	79,423 (42.2)
Geographical region**, n (%)		
North China	11,247 (11.7)	19,041 (10.1)
East China	59,749 (61.9)	120,677 (64.0)
Central China	944 (0.98)	1,735 (0.92)
South China	5,409 (5.60)	11,411 (6.06)
Northeast China	3,870 (4.01)	8,516 (4.52)
Northwest China	9,666 (10.01)	16,800 (8.92)
Southwest China	5,632 (5.84)	10,240 (5.43)

*According to the new standards in 2014, urban populations are more than 10 million for megacity behemoth, 5-10 million for mega city, 1-5 million for large cities (3-5 million for type I large cities, 1-3 million for type II large cities), 0.5-1 million for medium-sized cities, and less than 0.5 million for small cities (0.2-0.5 million for type I small cities, and less than 0.2 million for the type II small cities). The city size was divided according to the sixth census in 2010.

**Seven geographical regions of China: https://www.chinacheckup.com/blogs/articles/regions-of-china

†BMI was categorized according to Chinese guidelines: underweight (BMI<18.5 kg/m^2^), normal (18.5-23.9 kg/m^2^), overweight (24-27.9 kg/m^2^), and obesity (≥28 kg/m^2^).

**Figure 3:**
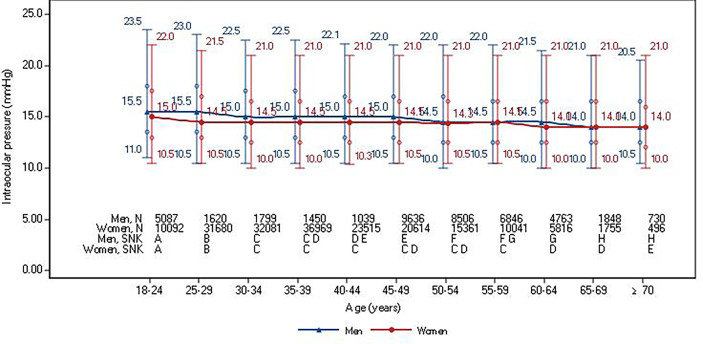
Median and 95% reference intervals of intraocular pressure in healthy participants according to sex and age stratifications. Circles denote interquartile ranges. Student-Newman-Keuls (SNK) test was used for multiple comparison of IOP levels among different age groups by gender and age. The same capital letters indicated different means of the index groups were equal, but different capital letters indicated different means of index groups were not equal.

The IOP showed a general trend of decline with age in both sexes (**Figure 3**). The median IOP of men decreased gradually from 15.5 (RI: 11.0-23.5) mmHg at ages 18-24 to 14.0 (RI: 10.5-20.5) mmHg at age ≥70 years. The median IOP of women decreased gradually from 15.0 (RI: 10.5-22.0) mmHg at ages 18-24 to 14.0 (RI: 10.0-21.0. mmHg at age ≥70 years. After the age of 65 years, the IOP of men was similar to that of women. According to the multiple comparison of IOP levels among different age groups using the SNK test in **Figure 3**, there was a significant difference between all the age groups of 18-24, 25-29, 30-34, 45-49, 50-54, 60-64, and ≥65 years in men and age groups–18-24, 25-29, 30-34, 60-64, and ≥70 years in women.

**Figure 4:**
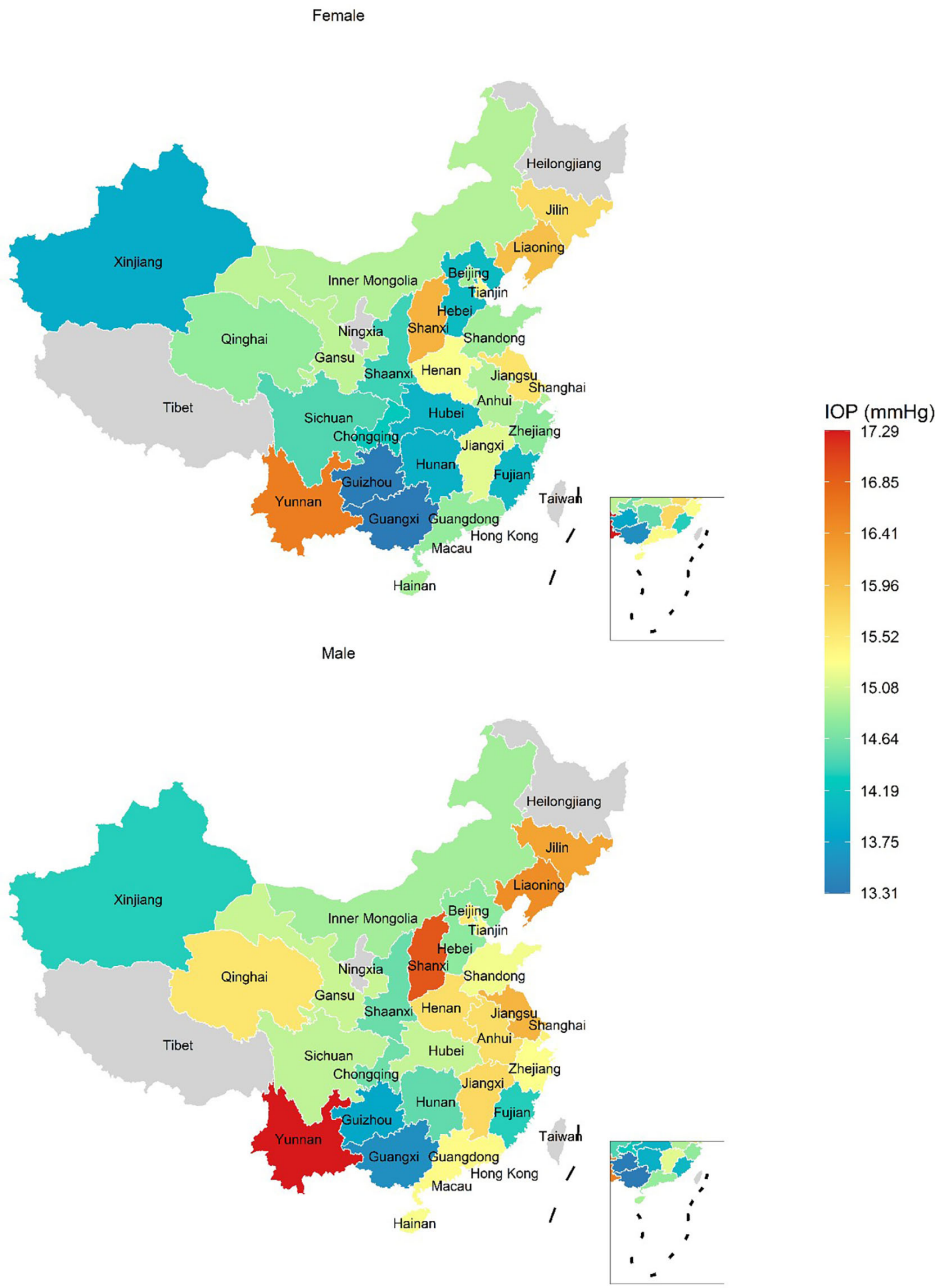
The adjusted mean of intraocular pressure in healthy participants. Intraocular pressure was adjusted for age and gross domestic product by a general linear model. Choropleth maps were produced within R software according to the decile of province intraocular pressure levels.

IOP levels varied across geographical locations. The age-GDP adjusted mean IOP is presented for both sexes in **Figure 4**. In men, the lowest standardized 95% reference interval was 9.0-22.5 mmHg in Guangxi and the highest 95% reference interval was 11.5-23.2 mmHg in Yunnan province. For women, the lowest standardized 95% reference interval was 8.7-20.0 mmHg in Guizhou province and the highest 95% reference interval was 11.2-21.5 mmHg in the Yunnan province.

### Stratification analysis results

Certain factors may influence IOP levels, such as height and blood pressure. We further calculated the IOP (RIs) in subgroups of height tertiles, normal or overweight, normal or prehypertension, altitude tertiles, and north or south geographic location for different age groups (**Table 2**). The results in the general population (participants with certain diseases) are also displayed (**Table S2**).

**Table 2:** Median and 95% reference interval of intraocular pressure of participants in the study.

Men					Women			
	All(N=96,517)	<30 years(N=21,292)	30-60 years(N=67,884)	≥60 years(N=7,341)	All (N=188,420)	<30 years(N=41,772)	30-60 years(N=138,581)	≥60 years(N=8,067)
Height*,cm								
Tertile 1	14.5(10.5-21.5)	16.0(11.0-22.5)	14.5(10.5-21.5)	14.0(10.0-21.5)	14.5(10.5-21.0)	15.0(10.5-22.0)	14.5(10.5-21.0)	14.0(10.0-21.0)
Tertile 2	14.5(10.5-22.0)	15.5(10.5-23.5)	14.5(10.5-22.0)	14.0(10.0-21.0)	14.5(10.5-21.5)	15.0(10.5-21.5)	14.5(10.5-21.0)	14.0(10.5-21.5)
Tertile 3	15.0(10.5-22.5)	15.5(10.5-23.0)	15.0(10.5-22.5)	14.5(10.0-21.5)	14.5(10.0-21.5)	15.0(10.5-22.0)	14.5(10.0-21.0)	14.5(10.5-20.5)
BMI^&^, kg/m^2^								
<24	14.5(10.5-21.5)	15.0(10.5-22.0)	14.5(10.3-21.3)	13.5(10.0-20.0)	14.5(10.0-21.5)	14.5(10.5-21.5)	14.0(10.0-21.0)	14.0(10.0-20.5)
24-27.9	15.0(10.5-22.3)	15.5(10.5-23.0)	14.5(10.5-22.0)	14.0(10.0-21.0)	14.5(10.5-21.0)	15.0(10.5-22.0)	14.5(10.1-21.0)	14.0(10.0-21.0)
Blood pressure								
Normal	14.5(10.5-22.0)	15.0(10.5-22.5)	14.5(10.5-21.5)	14.0(10.0-21.0)	14.5(10.0-21.0)	14.5(10.5-21.5)	14.0(10.0-21.0)	14.0(10.0-20.5)
Pre-hypertension	15.5(10.5-23.0)	16.0(11.0-24.0)	15.0(10.5-23.0)	14.5(10.5-21.5)	15.0(10.5-22.0)	15.5(11.0-23.0)	15.0(10.5-22.0)	14.5(10.5-21.0)
Altitude*, m								
Tertile 1	15.5(10.5-23.0)	16.0(10.5-23.7)	15.5(10.5-22.5)	14.5(10.5-22.0)	15.0(10.5-21.1)	15.0(10.5-22.5)	14.5(10.5-21.0)	14.5(10.5-21.0)
Tertile 2	14.5(10.5-22.5)	15.5(10.5-23.0)	14.5(10.0-22.0)	14.0(10.0-21.5)	14.0(10.0-21.0)	14.5(10.5-21.5)	14.0(10.0-21.0)	13.5(10.0-20.5)
Tertile 3	14.5(10.5-22.4)	15.0(11.0-23.0)	14.5(10.5-22.0)	14.0(10.0-21.0)	14.5(10.5-21.5)	14.5(10.5-22.0)	14.5(10.5-21.5)	14.0(10.5-21.5)
Geographic area†								
North	15.0(10.5-22.5)	15.0(11.0-23.0)	15.0(11.0-22.5)	14.5(10.5-21.5)	14.5(10.5-21.5)	14.5(10.5-22.0)	14.5(10.5-21.5)	14.5(10.5-21.5)
South	15.0(10.5-22.5)	15.5(10.5-23.0)	15.0(10.5-22.0)	14.0(10.0-21.5)	14.5(10.0-21.0)	15.0(10.5-22.0)	14.5(10.0-21.0)	14.0(10.0-21.0)

*Height and altitude are divided by tertiles.

^&^BMI: Body mass index.

†Geographic area divided by latitude of 33°N.

The IOP reference intervals varied with BMI (**Table 2**). The highest IOP levels were found in the higher BMI and younger age groups, while the lowest IOP levels were found in the lower BMI and older age groups in both sexes (**Table 2**). Higher BMI tended to have higher IOP in the same age group. In male participants, the highest IOP levels (15.5 (10.5-23.0) mmHg) were found in the overweight group and age <30 years, while the lowest IOP levels (13.5 (10.0-20.0) mm Hg) were found in those with BMI <24 kg/m^2^ and age ≥60 years. Similar results were observed in female participants.

pt?>IOP RIs varied with blood pressure (**Table 2**). The highest IOP levels were found in the higher blood pressure and younger age groups, while the lowest IOP levels were found in the lower blood pressure and older age groups in both sexes. Higher blood pressure tended to have higher IOP in the same age group. In male participants, the highest IOP levels (16.0 (11.0-24.0) mmHg) were found in those with the highest blood pressure and age <30 years, while the lowest IOP levels (14.5(10.5-21.5) mmHg) were found in those with the lowest blood pressure and age ≥60 years. Similar results were observed in female participants.

The IOP RIs varied with altitude (**Table 2**). The highest IOP levels were found in the lower altitude and younger age groups, while the lowest IOP levels were found in higher altitude and older age groups in both sexes. Lower altitude groups tended to have higher IOP in the same age group. In male participants, the highest IOP levels (16.0 (10.5-23.7) mmHg) were found in the group from the lowest altitude and with age <30 years, while the lowest IOP levels (14.0 (10.0-21.0) mm Hg) were found in groups from the highest altitude and with age ≥60 years.

However, IOP reference intervals did not show any differences by height tertiles and geographic area divided by latitude of 33° N in all three age groups.

## Discussion

To the best of our knowledge, the current study is the largest investigation using national data to examine nuanced IOP differences by sex, age, and geographic location in China. We found that the distribution of IOP varied with all three factors. BMI, blood pressure, and altitude also influenced IOP.

IOP is an important factor for glaucoma pathogenesis, and is also crucial for monitoring the treatment of glaucoma. Therefore, it is important to identify the variations in IOP by sex, age, and geographical location. Investigation of specific RIs of IOP will help to distinguish normal eyes from glaucomatous eyes based on IOP readings and contribute to setting precise IOP RIs for glaucoma treatments in diverse patients.

This study suggests that the association between sex and IOP may be modified by age. We found that below 60 years of age, men had a higher IOP than women. However, men and women over 60 years of age had similar IOP values. The association between sex and IOP was inconsistent in previous studies (6, 11). This could be due to the studies reporting no gender difference in IOP being performed with participants aged 55 years and over (6, 11), while others observing higher IOP values in men being performed in younger participants aged about 40 years (4). The gender difference may be influenced by hormonal regulation (5, 19). However, further research is warranted to better understand the mechanisms underlying sex differences in IOP.

The IOP showed a general trend of decline with age in both sexes. There are studies that show IOP increasing with age (6), decreasing with aging (7), and not associated with age after adjustment (20, 21). Several factors, such as a narrow age span, limited amounts of samples, and a lack of sex-specific analysis, may lead to inconsistent results. In our study, we found that the median IOP decreased from 18 years (15.5 mmHg) to 70 years of age and over (14.0 mmHg) in male participants. The general trend among women is similar to men.

The diverse IOP distribution in different locations has been reported in previous studies (2, 22–24). The IOP was reported as 17.1 ± 3.1 mmHg (22) in the Baltimore Eye Survey in the United States, 14.5 ± 2.6 mmHg in the Tehran Eye Study in Iran (23), and 12.9 ± 3.1 mmHg in the Shihpai eye study (24) in Shihpai, Taiwan. Here, the IOP distribution in China also varied from 15.6 ± 3.0 mmHg in Beijing to 15.3 ± 2.3 mmHg in Guangzhou (25). Previous spatially orientated research was limited by comparisons across singular and coarse geographic scales. In the current study, we were able to investigate the variation of IOP RIs across China, accounting for administrative regions, altitude, and latitude, by the data from 170 health screening centers covering 81 cities in mainland China. We found that the IOP values varied from 8.7-20.0 mmHg in Guizhou to 11.2-21.5 mmHg in Yunnan province. The study has shown that IOP levels are higher at lower altitudes for different genders and ages, with the exception that the highest IOP occurred in Yunnan Province, which has a higher average altitude. An investigation of IOP from a geographical perspective will provide the necessary information for allocation of health resources to prevent and control glaucoma.

Several studies have shown that IOP is influenced by risk factors in metabolic disorders such as diabetes (26), blood pressure levels (27),obesity (28), and dyslipidemia (29). We found that participants with certain metabolic disorders, such as being overweight and pre-hypertension, have higher IOP. This may indicate that IOP levels were influenced by diseases or risk factors of metabolic disorders. Another possible explanation is that obesity may affect breathing, which may influence the measurement of intraocular pressure when the non-contact IOP is usually measured in sitting posture.

The distribution of IOP varied with sex, age, and geographic location, suggesting that the variation in IOP should be corrected according to these factors in medical practice. The traditional normal range of IOP (10 to 21 mmHg) was based on a hospital-based study performed by Leydhecker and colleagues (12) in 1958 and an investigation conducted by Hollows and Graham in 1966 (10). These studies were limited to Caucasians, and the results were not stratified by sex, age, or geographic area. Hypertension, diabetes, obesity, and other metabolic disorders, which also can alter IOP dramatically, were not taken into account. Especially in the diagnosis of “normal tension glaucoma”, the “normal range of intraocular pressure” may need to be further precisely defined according to age, sex, metabolic disorders and geographic location.

The limitations of this study need to be acknowledged. First, IOP was measured using a non-contact tonometer, and the central corneal thickness was not taken into account in IOP measurements; however, the non-contact tonometers in our study have good quality control and are still widely used as a screening tool for glaucoma in China and other countries (30). Second, the participants of this study were mainly those selected for health screening, and thus do not represent the general population. However, the number of people undergoing health screening in China reached 575 million in 2018, accounting for 42% of its population in 2018. The results of this study may provide insights into this special population. Thirdly, the **Figure 4** indicated there were some IOP variations in certain ethnic minority areas, such as Yunnan and Xinjing. However, there was no available information on race for individuals in this real-world data. The influence of race on the variation in IOP should be further studied.

In summary, the IOP varied with age, sex, metabolic disorders and geographic location. These RIs should be considered in the clinical process of glaucoma diagnosis and treatment.

## Data availability statement

The raw data supporting the conclusions of this article will be made available by the authors, without undue reservation.

## Ethics statement

The studies involving human participants were reviewed and approved by The Ethics Committee of Beijing Tsinghua Changgung Hospital, affiliated with Tsinghua University (Ethics number 18181-0-01). The patients/participants provided their written informed consent to participate in this study.

## Author contributions

XL, XP, and YM wrote the main manuscript text, and prepared figures and tables. CJ, BW, and YN provided the data. All authors reviewed the manuscript.

## Funding

This work was supported by the National Natural Science Foundation of China (91846303) and the Ministry of Science and Technology of China (2020YFC2003400).

## Acknowledgments

We thank all study participants and appreciate the contributions made by all health professionals in Meinian health screening centers.

## Conflict of interest

The authors declare that the research was conducted in the absence of any commercial or financial relationships that could be construed as a potential conflict of interest.

## Publisher’s note

All claims expressed in this article are solely those of the authors and do not necessarily represent those of their affiliated organizations, or those of the publisher, the editors and the reviewers. Any product that may be evaluated in this article, or claim that may be made by its manufacturer, is not guaranteed or endorsed by the publisher.

## Supplementary material

Supplementary Table 1 Sociodemographic and clinical characteristics of the general population^‡^ (N=739,928) by sex

Supplementary Table 2 Median and 95% reference interval of intraocular pressure of the general population^‡^ according to sex and age and further stratified by height, body mass index, blood pressure, altitude, geographic area
