# Supplementary material for: Variation in intraocular pressure by sex, age, and geographic location in China: A nationwide study of 284,937 adults
Source: Front Endocrinol (Lausanne). 2022 Aug 25;13:949827. doi: 10.3389/fendo.2022.949827 (PMC9453811; doi:10.3389/fendo.2022.949827)
Supplement: Supplementary Table 1 — Sociodemographic and clinical characteristics of the general population‡ (N=739,928) by sex [file DataSheet_1.docx]

**Appendix**

**Table S1. Sociodemographic and clinical characteristics of the general population^‡^ (N=739,928) by sex**

|  |  | Men | Women |
| --- | --- | --- | --- |
| N |  | 376,230 | 363,698 |
| Age (years), Mean±SD |  | 42.32±12.31 | 42.65±12.26 |
| <30, n (%) |  | 60,951 (16.2) | 59,326 (16.3) |
| 30-40, n (%) |  | 116,987 (31.1) | 105,170 (28.9) |
| 40-50, n (%) |  | 87,236 (23.2) | 85,106 (23.4) |
| >=50, n (%) |  | 111,056 (29.5) | 114,096 (31.4) |
| IOP (mmHg), Mean±SD |  | 15.74±3.33 | 15.06±3.08 |
| Right IOP |  | 15.65±3.51 | 14.98±3.24 |
| Left IOP |  | 15.84±3.53 | 15.13±3.27 |
| Gross Domestic Product per capita (CNY), n (%) |  |  |  |
| Quintiles 1 |  | 80,992 (21.5) | 68,093 (18.7) |
| Quintiles 2 |  | 75,900 (20.2) | 73,814 (20.3) |
| Quintiles 3 |  | 100,345 (26.7) | 119,211 (32.8) |
| Quintiles 4 |  | 46,111 (12.3) | 28,132 (7.73) |
| Quintiles 5 |  | 72,882 (19.4) | 74,448 (20.5) |
| Hypertension, n (%) |  |  |  |
| Normal |  | 129,989 (35.1) | 203,073 (57.0) |
| Prehypertension |  | 145,287 (39.2) | 95,631 (26.8) |
| Hypertension |  | 95,016 (25.7) | 57,682 (16.2) |
| Diabetes, n (%) |  |  |  |
| Normal |  | 57,108 (61.7) | 59,213 (71.7) |
| Prediabetes |  | 25,969 (28.1) | 18,729 (22.7) |
| Diabetes |  | 9,481 (10.2) | 4,628 (5.60) |
| BMI^†^ (kg/m^2^), Mean±SD |  | 24.95±3.45 | 23.02±3.36 |
| <18.5, n (%) |  | 8,112 (2.22) | 21,429 (6.10) |
| <24, n (%) |  | 138,056 (37.8) | 209,332 (59.6) |
| <28, n (%) |  | 156,139 (42.8) | 92,644 (26.4) |
| >=28, n (%) |  | 62,684 (17.2) | 27,674 (7.88) |
| City size*, n (%) |  |  |  |
| Small or medium sized city |  | 52,034 (13.8) | 57,987 (15.9) |
| Large city |  | 159,614 (42.4) | 152,230 (41.9) |
| Mega city and above |  | 164,582 (43.7) | 153,481 (42.2) |
| Geographical region**, n (%) |  |  |  |
| North China |  | 48,705 (12.9) | 38,834 (10.7) |
| East China |  | 224,723 (59.7) | 229,471 (63.1) |
| Central China |  | 4,693 (1.25) | 3,824 (1.05) |
| South China |  | 23,938 (6.36) | 22,584 (6.21) |
| Northeast China |  | 17,477 (4.65) | 19,375 (5.33) |
| Northwest China |  | 37,090 (9.86) | 31,993 (8.80) |
| Southwest China |  | 19,604 (5.21) | 17,617 (4.84) |

‡ Including participants with certain major diseases, including cardio-cerebro-vascular disease, hypertension, diabetes, dyslipidemia, obesity, and hyperuricemia, osteoporosis, anemia, ALT >=40 U/L, severe chronic kidney disease (eGFR <30 mL/min/1.73 m^2^ or proteinuria).

* According to the new standards in 2014, urban populations are more than 10 million for megacity behemoth, 5 – 10 million for mega city, 1–5 million for large cities (3–5 million for type I large cities, 1 - 3 million for type II large cities), 0.5 1 million for medium-sized cities, less than 0.5 million for small cities (0.2 0.5 million for type I small cities, less than 0.2 million for type II small cities).The city size was divided according to the sixth census in 2010.

**Seven geographical regions of China:

https://www.chinacheckup.com/blogs/articles/regions-of-china

† BMI was categorized according to Chinese guidelines: underweight (BMI <18.5 kg/m^2^), normal (18.5-23.9 kg/m^2^), overweight (24-27.9 kg/m^2^), and obesity ( ≥28 kg/m^2^).

**Table S2. Median and 95% reference interval of intraocular pressure of the general population^‡^ according to sex and age and further stratified by height, body mass index, blood pressure, altitude, geographic area**

|  |  | Men | | |  |  | Women | | |
| --- | --- | --- | --- | --- | --- | --- | --- | --- | --- |
|  | All  (N=376,230) | <30 years  (N=60,951) | 30-60 years  (N=276,427) | ≥60 years  (N=38,852) |  | All  (N=363,698) | <30 years  (N=59,326) | 30-60 years  (N=266,584) | ≥60 years  (N=37,788) |
| Height*, cm |  |  |  |  |  |  |  |  |  |
| Tertile 1 | 15.0(10.5-22.5) | 15.6(11.0-24.0) | 15.0(10.5-22.5) | 14.5(10.0-22.0) |  | 14.5(10.5-22.0) | 15.0(10.5-22.0) | 14.5(10.5-21.5) | 14.5(10.5-22.0) |
| Tertile 2 | 15.0(10.5-23.0) | 15.5(11.0-23.5) | 15.0(10.5-23.0) | 14.5(10.5-22.5) |  | 14.5(10.5-22.0) | 15.0(10.5-22.0) | 14.5(10.5-21.5) | 14.8(10.5-22.0) |
| Tertile 3 | 15.5(10.5-23.5) | 15.5(11.0-23.5) | 15.5(10.5-23.5) | 15.0(10.5-23.0) |  | 15.0(10.5-22.0) | 15.0(10.5-22.5) | 14.5(10.0-22.0) | 15.0(10.5-21.0) |
| Body mass index, kg/m^2^ |  |  |  |  |  |  |  |  |  |
| <24 | 14.5(10.0-22.0) | 15.0(10.5-22.0) | 14.5(10.0-22.0) | 13.5(10.0-20.5) |  | 14.5(10.0-21.5) | 14.5(10.5-21.5) | 14.5(10.0-21.0) | 14.0(10.0-20.5) |
| 24-27.9 | 15.0(10.5-22.5) | 15.5(10.5-23.0) | 15.0(10.5-22.5) | 14.5(10.2-22.0) |  | 14.5(10.5-21.5) | 15.0(10.5-22.0) | 14.5(10.5-21.5) | 14.5(10.5-21.5) |
| >=28 | 15.5(10.5-23.5) | 16.0(11.0-24.0) | 15.5(10.7-23.5) | 15.0(10.5-23.0) |  | 15.0(10.5-22.0) | 15.5(10.5-23.0) | 15.0(10.5-22.0) | 14.5(10.5-22.0) |
| Blood pressure |  |  |  |  |  |  |  |  |  |
| Normal | 14.5(10.5-22.0) | 15.0(10.5-22.5) | 14.5(10.5-22.0) | 14.0(10.0-21.0) |  | 14.5(10.0-21.0) | 14.5(10.5-21.5) | 14.0(10.0-21.0) | 14.0(10.0-20.5) |
| Pre-hypertension | 15.5(11.0-23.5) | 16.0(11.0-24.0) | 15.5(11.0-23.5) | 14.5(10.5-22.0) |  | 15.0(10.5-22.5) | 15.5(11.0-23.5) | 15.0(10.5-22.0) | 14.5(10.5-21.0) |
| Hypertension | 16.0(11.0-24.5) | 17.0(11.0-25.0) | 16.0(11.0-24.5) | 15.0(10.5-23.0) |  | 15.5(11.0-23.0) | 16.0(11.0-24.5) | 15.5(11.0-23.2) | 15.0(10.5-22.5) |
| Altitude*, m |  |  |  |  |  |  |  |  |  |
| Tertile 1 | 16.0(10.5-24.0) | 16.0(11.0-24.0) | 16.0(10.5-24.0) | 15.0(10.5-23.0) |  | 15.0(10.5-22.0) | 15.5(10.5-23.0) | 15.0(10.5-21.5) | 15.0(10.5-21.5) |
| Tertile 2 | 15.0(10.5-23.0) | 15.5(10.5-23.5) | 15.0(10.5-23.0) | 14.0(10.0-22.0) |  | 14.5(10.0-21.5) | 14.5(10.5-22.0) | 14.0(10.0-21.5) | 14.0(10.0-21.0) |
| Tertile 3 | 15.0(11.0-23.0) | 15.5(11.0-23.0) | 15.2(11.0-23.0) | 14.5(10.5-22.0) |  | 14.5(10.5-22.0) | 14.5(10.5-22.0) | 14.5(10.5-22.0) | 14.5(10.5-22.5) |
| Geographic area† |  |  |  |  |  |  |  |  |  |
| North | 15.5(11.0-23.5) | 15.5(11.0-23.5) | 15.5(11.0-23.5) | 15.0(10.5-22.5) |  | 15.0(10.5-22.5) | 14.5(10.5-22.0) | 15.0(10.5-22.5) | 15.0(10.5-22.5) |
| South | 15.5(10.5-23.1) | 16.0(10.5-23.5) | 15.5(10.5-23.0) | 14.5(10.5-22.5) |  | 14.5(10.5-21.5) | 15.0(10.5-22.0) | 14.5(10.5-21.5) | 14.5(10.5-21.5) |

‡ Including participants with certain major diseases, including cardio-cerebro-vascular disease, hypertension, diabetes, dyslipidemia, obesity, and hyperuricemia, osteoporosis, anemia, ALT >=40 U/L, severe chronic kidney disease (eGFR <30 mL/min/1.73 m^2^ or proteinuria.

*Height and altitude are divided by tertiles.

†Geographic area divided by latitude of 33°N
